# A Comprehensive Analysis of the T and B Lymphocytes Repertoire Shaped by HIV Vaccines

**DOI:** 10.3389/fimmu.2018.02194

**Published:** 2018-09-26

**Authors:** Longlong Wang, Wei Zhang, Liya Lin, Xiao Li, Nitin K. Saksena, Jinghua Wu, Shiyu Wang, Joseph G. Joyce, Xiuqing Zhang, Huanming Yang, Jian Wang, I-Ming Wang, Xiao Liu

**Affiliations:** ^1^BGI-Education Center, University of Chinese Academy of Sciences, Shenzhen, China; ^2^BGI-Shenzhen, Shenzhen, China; ^3^China National GeneBank, BGI-Shenzhen, Shenzhen, China; ^4^Merck & Co., Inc., Kenilworth, NJ, United States; ^5^James D. Watson Institute of Genome Sciences, Hangzhou, China

**Keywords:** HIV, vaccine, T cell receptors repertoire, B cell receptors repertoire, gp41

## Abstract

The exploitation of various human immunodeficiency virus type-1 (HIV-1) vaccines has posed great challenges for the researchers in precisely evaluating the vaccine-induced immune responses, however, the understanding of vaccination response suffers from the lack of unbiased characterization of the immune landscape. The rapid development of high throughput sequencing (HTS) makes it possible to scrutinize the extremely complicated immunological responses during vaccination. In the current study, three vaccines, namely N36, N51, and 5-Helix based on the HIV-1 gp41 pre-hairpin fusion intermediate were applied in rhesus macaques. We assessed the longitudinal vaccine responses using HTS, which delineated the evolutionary features of both T cell and B cell receptor repertoires with extreme diversities. Upon vaccination, we unexpectedly found significant discrepancies in the landscapes of T-cell and B-cell repertoires, together with the detection of significant class switching and the lineage expansion of the B cell receptor or immunoglobulin heavy chain (IGH) repertoire. The vaccine-induced expansions of lineages were further evaluated for mutation rate, lineage abundance, and lineage size features in their IGH repertoires. Collectively, these findings conclude that the N51 vaccine displayed superior performance in inducing the class-switch of B cell isotypes and promoting mutations of IgM B cells. In addition, the systematic HTS analysis of the immune repertoires demonstrates its wide applicability in enhancing the understanding of immunologic changes during pathogen challenge, and will guide the development, evaluation, and exploitation of new generation of diagnostic markers, immunotherapies, and vaccine strategies.

## Introduction

After 36 years since HIV was discovered, conquering HIV-1 still remains a challenge for mankind. The development of a safe and highly effective vaccine against HIV is challenging because of high genetic variability HIV displays which assists HIV to evade host immune responses. The main challenge also lies in that the immune-protective correlates of HIV/AIDS remain unknown, and that HIV acquires resistance to all known classes of antiretroviral drugs currently prescribed. As none of the vaccines have succeeded, to date, in controlling HIV, the Highly Active Anti-Retroviral therapy (HAART) remains the hallmark of treatment for HIV patients. Drugs such as fusion, protease, reverse transcriptase, and integrase inhibitors (1, 2) can block the virus life cycle at different steps of infection process. Even though these drugs provide a good quality life and have decreased the rate of mortality in HIV patients, they are marred by the development of drug resistance in HIV patients. This leaves the vaccination-augmented immune system a prospective and cost-effective alternative to eliminate the infection. Unfortunately, none of the anti-HIV vaccine strategies, to date, have worked.

Surface envelope glycoproteins gp120 and gp41 of HIV-1, are critical for viral entry. For this, HIV-1 gp120 protein firstly interacts with CD4 receptor, followed by engagement with co-receptors CCR5 or CXCR4 on T cells triggering the conformational change in the transmembrane gp41 molecule thereby facilitating viral and cellular membrane fusion leading to viral entry into the host cell (3). Thus, due to their integral role in viral both gp120 and gp41 have been the major targets for HIV vaccine development (3). For example, Enfuvirtide, a 36 residue synthetic peptide that binds to gp41 trimer prevents the formation of the membrane fusion and viral entry, demonstrated clinical success, and was approved by the United States Food and Drug Administration(USFDA) in 2003 (4). Thus, developing vaccines that can induce gp41-neutralizing antibodies, and potentially function in analogous way as Enfuvirtide, has attracted considerable interest.

Some patients develop bNAbs for HIV viruses after several years of infection (5–8). Studies of monoclonal bNAbs, such as 2F5, 4E10, and Z13e, have identified the viral neutralization epitopes of gp41 on either N or C terminal (9–11). Thus, the primary focus of the vaccination is to induce monoclonal antibodies (mAbs) which can bind to gp41 N and C–terminal heptad region (NHR and CHR, respectively) and perturb the trimer association. In fact, the gp41 NHR gets exposed only when gp120 interacts with CD4 and co-receptors, forming a transient conformation of gp41 termed “pre-hairpin” intermediate. One example to illustrate this mechanism is that the DP178 peptide can only associate with the gp41 leucine/isoleucine zipper sequence at this transient intermediate status instead of the six-helical bundled three hairpin structure after the gp41 conformation changes (12).

In order to induce the antibody (Ab) targeting the pre-hairpin intermediate, the vaccine must stably preserve the correct assembly structure while remaining *in vivo*. The IQN17 is a soluble trimeric 17-mer peptide which mimics the NHR region of the pre-hairpin intermediate designed by Eckert et al. for further development and identification of anti-HIV drugs, and it was proved to be the same structure as is present in the actual pre-hairpin intermediate status (13). Based on the structure of IQN17, many vaccines were designed and tested. Bianchi et al. demonstrated that prolonging the antibody binding site outside the hydrophobic pocket region on the peptide could strengthen the neutralization efficiency of antibodies (Abs) induced by the vaccine. The stability and length optimized trimer protein-N36 which exhibits mimicry to the IQN17 protein showed encouraging performance as a vaccine (14). Besides, an even longer N51 trimer protein encompassing the entire gp41 NHR presents new neutralizing determinants distinct from the traditional IQN17 D5 epitope, which is quite enlightening in vaccine development. The 5-Helix vaccine, unlike the two NHR-based vaccine–N36 and N51, was based on the structure of the gp41 6-helix structure after the NHR and CHR association: one of the three C-peptide helices was removed to create a vacancy for the high gp41 C-terminal affinity region, while the other five helices were connected with short peptide linkers (11, 15). It was speculated that a 5-Helix–like intermediate may firstly form before the accomplishment of the 6-helix fusion process so that it may act as a potential vaccine candidate (16). Abs which can combine with the 5-Helix-like intermediate will probably hinder the formation of the 6-helix structure, thus prevent the viral entrance. In this study, the three different peptides N36, N51, and 5-Helix were modified as the vaccines to immunize the macaques.

Complementarity determining region 3 (CDR3) determines the T cell antigen recognition specificity. CDR3 is generated through a series of recombination of noncontiguous variable (V), diversity (D), and joining (J) region gene segments. Deletion and insertion of nucleotides at the Vβ-Dβ and Dβ-Jβ junction during the rearrangement further increase the emerging frequencies of the high antigen affinity T cell receptor beta (TCRB). Similarly, B cells produce B cell receptor (BCR) and immunoglobulins through the rearrangement of the V, D, J gene loci. Somatic hypermutations and class-switching are introduced during the affinity maturation of B cells. B cell CDR3 largely determines the antibody affinity and is responsible for the tremendous diversity of the antibody repertoires in human (17).

The traditional way to monitor the vaccine responses was serological measurements which only provides imprecise estimation with limited molecular underpinnings. The high-throughput sequencing (HTS) has changed the way we look at immune repertoires and vaccine design (18). The HTS has provided many useful insights into mechanisms of immune responses through repertoire analysis (19–21), along with its potential clinical applications in the identification of new generation of biomarkers (22), therapeutic antibody design (23), and future design of anti-HIV vaccines. The fast development of HTS provides us with an invaluable opportunity to scrutinize the vast amount of information contained in the T and B cells populations. In fact, the adaptive immune response selectivity is based on the ever-changing diversity seen in T and B cell antigen-specific receptors. This immune repertoire, which is the collection of T and B cells is empowered with dynamic functional diversity in the circulatory system at any given time, and reflects the intrinsic nature of the immune selectivity. Here, we used *IMonitor* to further refine the massively paralleled sequencing data into the TCRB and IGH repertoire information (24). Rhesus macaques have been widely used as a model for HIV vaccines, along with their use in preclinical assessment of human vaccines (25), the rhesus Ig germline gene segment annotation, and the availability of relevant methods to phenotype and sort rhesus macaque B cell populations has made it possible to dissect vaccine- and infection-induced B cell responses.

HTS of B-cell repertoires offers a robust quantitative tool to analyze B-cell responses to any vaccine. In this study, the dynamics exhibited in the rhesus macaque TCRB and IGH repertoires induced by the three gp41-targeting vaccines, N36, N51, and 5-Helix, were evaluated. We delineated the vaccine boost process in detail and gained insights in the comparison of three vaccine groups. The methodology of sequencing data analysis provides novel alternatives for a traditional vaccine study.

## Materials and Methods

### Sample Collection and RNA Extraction

A total of 18 rhesus macaques (Indian Rhesus, *Macaca mulatta*) were collected for this study. Information of each macaque is described in detail in Supplementary Table 1. The macaques were divided randomly into 3 groups (5-Helix/N51/N36) according to the vaccine regimen used for immunization. The core epitope amino acid sequences of gp41 for the three vaccines were introduced previously (12, 16), the specific sequences are as follows:

N36:SGIVQQQNNLLRAIEAQQHLLQLTVWGIKQLQARIL

N51: QARQLLSGIVQQQNNLLRAIEAQQHLLQLTVWGIKQLQARILAVERYLKDQ

5-Helix-N40: QLLSGIVQQQNNLLRAIEAQQHLLQLTVWGIKQLQARILA

5-Helix-C38: HTTWMEWDREINNYTSLIHSLIEESQNQQEKNEQELLE

See also Supplementary Table 2.

**Figure 1:**
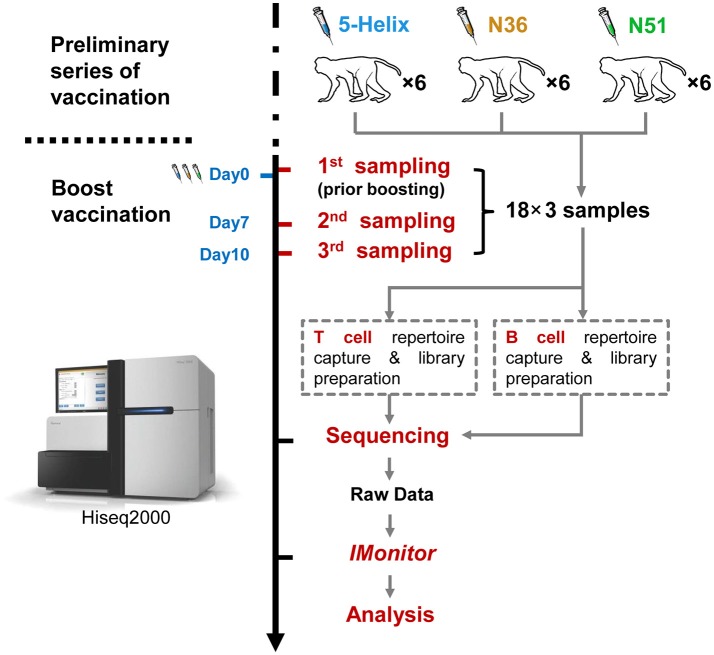
Schema of the work flow used in this study. Eighteen (18) rhesus macaques were divided equally into three groups (5-Helix, N51. and N36) (total 54 samples) and administered with the corresponding vaccines. The vaccination regimen contains the preliminary series of vaccination and the boost vaccination. The 18 macaques were sampled for the first time at Day 0 prior boost. After the boost, the macaques were further sampled at day 7 and 10. Both T and B cell repertoires of the 54 samples were captured, and prepared into 108 sequencing libraries for Illumina Hiseq2000. Raw sequencing data were then subjected to *IMonitor* work flow for basic TCR and IGH repertoire analysis.

In the beginning, a preliminary vaccination consisted of 4 vaccinations over a period of 36 weeks had been taken. Then, at the 66 week post-final vaccination of the preliminary vaccination, all the monkeys were boosted by the same vaccines, and altogether 54 peripheral blood samples were obtained at three time points during the boost vaccination stage, the overall strategy is described in Figure 1. The Peripheral blood mononuclear cells (PBMC) were immediately isolated from each sample using Ficoll-Paque gradient centrifugation (GE Healthcare). RNA was extracted from the PBMCs using Trizol reagent (Invitrogen,15596-026).

### Library Preparation and Repertoire Sequencing

Six hundred nanograms of total RNA was used for library preparation by 5′RACE kit (Invitrogen, 18374-058) as previously described (26). Complementary DNA (cDNA) was synthesized with SuperScript II reverse transcriptase (Invitrogen) by an Abridged Anchor primer (AAP) upstream and the IGH/TRB gene-specific primers downstream labeled with biotin. The gene specific primers were flanked with a PacI restriction enzyme recognition site of TTAATTAA, and also four protective bases of ACAC at the beginning of the 5′ site.

For analyzing the TRB repertoire, the gene-specific primer used for cDNA synthetize was:

5′-Bio ACACTTAATTAACTGGGAACACYTTTTTCAGGT-3′.

For analyzing the IgH repertoire, five primers of C gene were used:

5′-Bio ACACTTAATTAACCTSGGAGGTGCTCCTGGA-3′ (IgG),

5′-BioACACTTAATTAACGCACGCTGATATGATGGGG-3′ (IgD),

5′-Bio ACACTTAATTAACAGACCTTGGGKYTGGTCGG-3′ (IgA),

5′-Bio ACACTTAATTAACGTTGGGGCGGATGCACTC-3′ (IgM),

5′-BioACACTTAATTAACACGAAGGGGCTCTGTATG-3′ (IgE).

Following reverse transcription and template switching, PCR was performed corresponding to a forward primer annealed to the AAP region and a mixture of reverse primer annealed to the C region of TRB/IGH. The PCR program began with an initial denaturation at 94°C for 2 min, followed by 30 cycles of denaturation at 94°C for 30 s, annealing of primer to DNA at 60°C for 30 s, and extension by high fidelity Platinum Taq DNA Polymerase enzyme (Invitrogen) at 72°C for 45 s. Following the cycling, the reaction was at 72°C for 7 min, with final step at 4°C to hold the terminated reaction. The PCR products were purified by Ampure XP beads (Agencourt), and then fragmented into 150–200 bp using Covaris E220. M270 beads (Invitrogen), these were then utilized to capture the biotinylated C region with streptavidin. The biotin was lowered off by PacI restriction enzyme after recovering from M270 beads. Then the fragmented products were end repaired, adenine added at the 3′ site, ligated with Illumina adapters and amplified for 15 cycles with common primers. The sequencing libraries were quantified using Agilent 2100 Bioanalyzer system with DNA 12,000 kit and Applied Biosystems QPCR Bioanalyzer system, and sequenced by Hiseq2000 (Illumina) using paired-end 150 bp. All immune-sequencing data underlying this study are freely available from the PIRD Database project in China National Genebank website https://db.cngb.org/pird/project/P18080101/.

### Data Analysis

The sequencing data were then analyzed with *IMonitor* (24), which can be briefly described as follows. Firstly, the basic QC and low-quality reads filtration of the sequencing data and then merging of the cleaned paired-end reads was performed. Secondly, the BLAST (27–29) alignment of the merged data to the V, D, J germline genes and alleles. Subsequently, a re-alignment for each result was performed with a goal to select the best gene. Thirdly, the filtration of low abundance sequences with a less than 5 support reads number and then translate these nucleotide sequences into amino acid sequences. Fourthly, the statistics of TCR or IGH data, such as V-J pairing, V/J usage, CDR3 sequence frequency and CDR3 length distribution. The sequence number that were filtrated at different steps of IMonitor pipeline are appended in Supplementary Tables 3, 4.

The parameters of IMonitor used in these samples were: -d -k 150 -c -jif 70 -vif 70 -ec. For example, the TRB analysis in this manuscript was as follows: perl IMonitor.pl -a < 1fq.gz> -b < 2.fq.gz> -A1 < 1.adapter.list.gz> -A2 < 2.adapter.list.gz> -o < output filename>-n < sample id> -t TRB -d -k 150 -r <TRB reference file> -c -jif 70 -vif 70 -ec, where -a and -b are parameters for the two paired-ended raw data, -A1 and -A2 for the Illumina sequencing adaptor list, -o for output filename, -n for sample id, -t for gene type, e.g., TRB,IGH, -d for considering D genes in analysis, -k for read length, -r for the reference directory,-c for find CDR3 by conservative region, -ec for sequencing error correction in CDR3.-jif and -vif for cut off of low identity sequence alignment with the V and J gene. The subsequent analyses data were mainly based on the <^*^.structure.gz> file. The sequence number that were filtrated at different steps of IMonitor pipeline is attached in Supplementary Tables 3, 4.

It is noteworthy that the incompleteness of the Rhesus macaque IGH germline gene hindered the accuracy of subsequent analyses. Therefore, a refined version of the IGH germline gene reference dataset was used to overcome this problem. The new VH gene reference dataset was the combination of 23 IMGT database rhesus VH germline, 61 VH alleles shown in a previous study (30) and the 91 VH alleles predicted by our group (31) through IMPre (32), and the new JH gene reference dataset which comprised of 8 IMGT JH alleles and 13 novel JH gene predicted by IMPre. The reference dataset, which was aimed at identifying novel Rhesus macaque germline gene, can be obtained from our and another research group.

### The Shannon Index Calculation

The Shannon–Weiner index (33), has been used as indicator of immunological diversity in several previous studies (34). The lymphocytes clones in immune repertoire can analogically treated as the species in ecology where Shannon–Weiner index has already been widely used. The index is calculated as follows:

(1) $$\begin{array}{r} Shannon\;Index=-\overset{S}{\underset{i\;=\;1}{\sum }}p\left(i\right)lnp\left(i\right), \end{array}$$

where S denotes the total number of unique CDR3, and p(i) denotes the frequency of CDR3.

### Selection Criteria of Expanded TCR Clones and IGH Lineages

**Figure 3:**
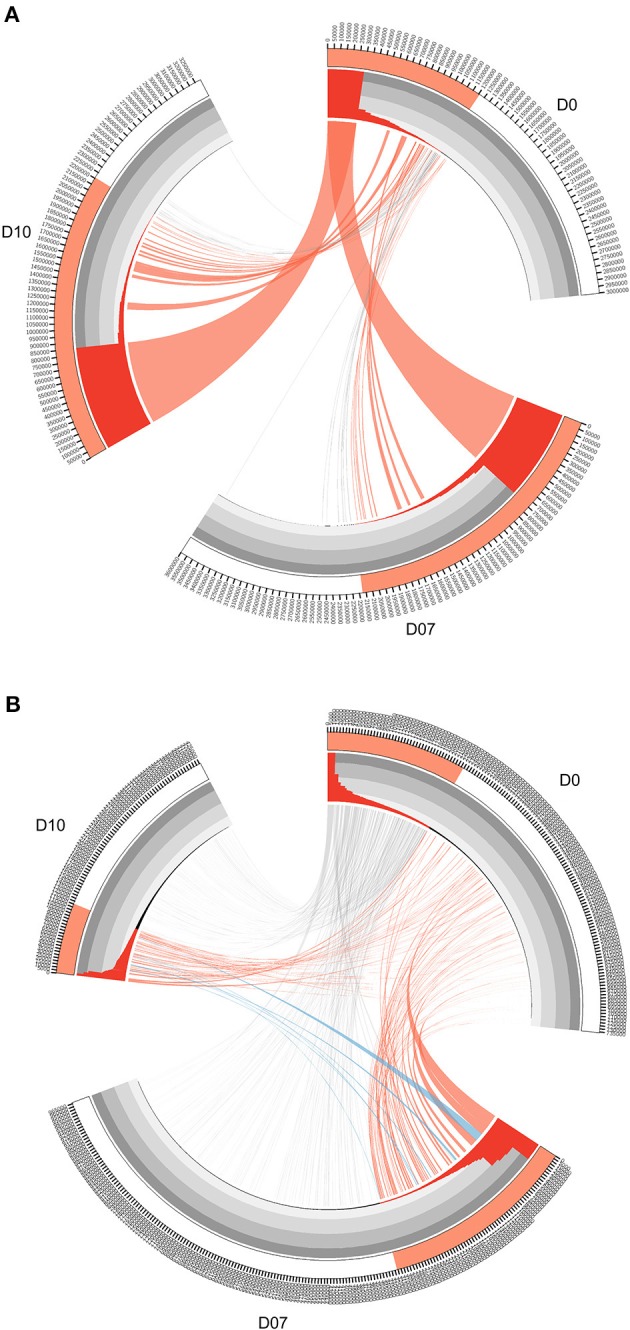
Circos diagrams of pattern change of TRB clone frequency and IGH lineage frequency during the time course of final boost of rhesus macaques A5R069 from the N51 group. **(A)** Clones were sorted by their frequency at three different time points, the clone number of each time point is ticked on the outer lane. High abundance clones (>0.01%) are marked in red. The inner histogram lane shows the frequency of each clone with the height of each bar representing the relative abundance. The width of both the histogram and ribbon represents the frequency of each clone. Ribbons connect the same TCR clones at different time points. The ribbons connecting the high abundance clones at D7 and D10 that showed increase in frequency of more than 1-fold compared to D0 were labeled in red, those connecting the clones showing 50% decrease in frequency were labeled in gray, and those connecting the newly emerged clones that have high abundance (>0.01%) were labeled in blue **(B)**. IGH lineages were plotted similarly as in **(A)**, with the exception that red ribbons connecting high abundance lineages (>0.05%) at D7 and D10 that showed increase in frequency by more than 3-fold compared to D0.

The TCR clones and IGH lineages that expanded specifically after the vaccination boost were considered to be potentially vaccine-induced. To filter for the most significant candidates, criteria based on the clone abundance was set as follows. First, the TCR clones with frequency expansion more than 1-fold at D7 or D10 were retained (corresponding to red ribbons in Figure 3). Second, the newly emerged high frequency (>0.01%) clones at both D7 and D10 that were originally undetectable at D0 were also retained (corresponding to blue ribbons in Figure 3). These two groups of the TCR clones were combined as vaccination-induced, or “expanded” clones and were subjected to further analysis. IGH lineages were defined as the sequences that were originated from the same VDJ recombination with no more than 1 amino acid difference in their CDR3 region (35). IGH lineages with frequency expansion more than 3-fold at D7 or D10, as well as the newly emerged high frequency (>0.05%) lineages at both D7 and D10 were similarly selected as vaccination-induced, or “expanded” IGH lineages. Specific expanded TCR and IGH clone information are listed in Supplementary Tables 5, 6.

### Statistics Analysis

In this study, paired or unpaired Mann–Whitney two-tailed test (by Wilcoxon test in R) was used where appropriate to compare groups and calculate the *p* value. *P* values were corrected by False Discovery Rate (FDR) for multiple tests. Correlations were calculated with Pearson correlation coefficients. Correlations were graded as low (r values between (0.2–0.39), moderate (0.4–0.59), strong (0.6–0.79), and very strong (≥0.8).

## Result

### TCR and IGH Repertoires Alteration Upon Vaccination

Three vaccines, including a trimeric 36-mer peptide “N36,” a 51-mer trimeric peptide “N51” and a recombinant pre-hairpin intermediate mimetic “5-Helix” were repeatedly administered to three groups of rhesus macaques with 6 individuals in each group (see Methods). As a panel of neutralization assays were performed during the preliminary vaccination stage, which showed a clear trend of improved potency where N51>N36>5-Helix (manuscript in preparation). We therefore wish to explore the reactions upon vaccine boost through immune repertoire sequencing and analysis, thus to help us to figure out what exactly had happened both in TCR and IGH repertoire.

Blood samples were collected at three time points during the boost vaccination stage: prior the boost (D0), 7 days (D7), and 10 days (D10) after the boost. Both TCR and IGH repertoires from all samples were subsequently sequenced and processed with the immune repertoire analysis software *IMonitor* (24) (Figure 1). After acquiring the CDR3 sequence statistics from *IMonitor*, we normalized each of the redundant TCR or IGH clones to a unanimous number. By doing so, we were able to eliminate the influence of data size on clonal diversity. We obtained four parameters through calculating the CDR3 clones to evaluate TCR and IGH repertoire, namely, unique CDR3 number, Shannon index, high-frequency clone number(HCN: frequency >0.1% clone number) and cumulate frequency of top100 clones with the highest frequency (CF100).

**Figure 2:**
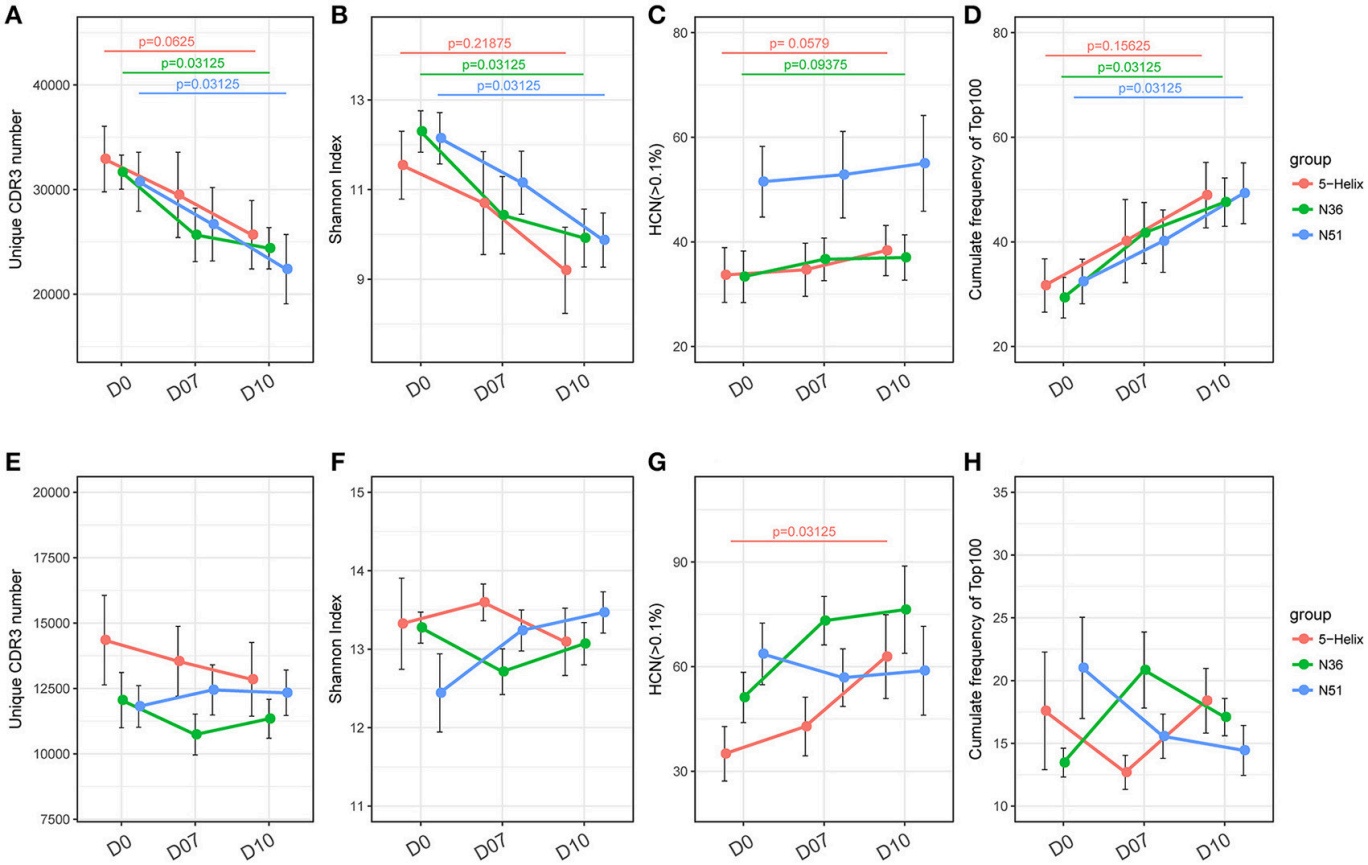
Overall response of vaccination on TCR and IGH repertoires. **(A–D)** Unique CDR3 numbers, Shannon index, high frequency (>0.1%) clone numbers and the cumulative frequency of the top 100 clones of the TCR repertoire. Each point represents the mean value of a 6-macaque group at one time point with a bar of ± SEM. Red, green, and blue color represents 5-Helix, N36, and N51, respectively. Comparisons were performed using paired Wilcoxon test (*n* = 6) **(E–H)**. Unique CDR3 numbers, Shannon index, high-frequency (>0.1%) clone numbers and the cumulative frequency of the top 100 clones of the IGH repertoire. Each point represents the mean value of a 6-macaque group at one time point with a bar of ± SEM. Red, green, and blue color represents 5-Helix, N36, and N51, respectively. Comparisons were performed using paired Wilcoxon test (*n* = 6).

Gradual decreases of the unique TCR CDR3 number and Shannon index in the three vaccination groups were observed at D7 and D10 (Figures 2A,B). Meanwhile, the CF100 of TCR evidently increased after the vaccination (Figure 2D). These results indicate that all three vaccines can significantly skew the repertoire toward the high frequency TCR clones, but it was not due to the expansion of low frequency clones because HCN was relatively stable (Figure 2C). It is more likely that the loss of TCR repertoire diversity was due to the expansion of the high frequency clones, especially the top100 abundant clones. Surprisingly, the IGH repertoires did not shift substantially in the four indices of IGH repertoire, implying a different mode of lineage expansion in the B-cells (Figures 2E–H).

### Discrepant Expansion Patterns of TCR and IGH Repertoires

The overall changes observed in the TCR repertoire (Figures 2A–D) could be partially explained by the dominant increase of certain high frequency clones after the vaccination boost. In order to find the origin and dynamics of expanded clones after the booster shot, we drew Circos diagrams to trace the clones and their frequencies in different time slots for each individual. Circos diagrams were constructed to exemplify the frequency and changes in the TCR repertoire constitution in rhesus macaques A5R069 from the N51 vaccination group (Figure 3A). Highlighted by the colored ribbon, high frequency clones at D7 and D10 were almost exclusively derived from existing highly abundant clones at D0. Very few high frequency clones at D0 suffered more than 50% decrease in frequency at D7 and D10. Other individuals in the same group and in other vaccine groups revealed a similar pattern (Supplementary Figure 2).

Interestingly, the IGH repertoire displayed a completely different pattern after vaccine boosting. As an example, in the N51 vaccination group, most of the novel high-frequency (>0.05%) clones at D7 and/or D10 were derived from the low frequency clones at D0. The frequencies of these clones expanded tens to hundreds of times and constituted a great portion of the most abundant lineages at D7 and D10. Conversely, the high frequency clones at D0 diminished drastically, except for very few clones maintaining comparable levels to D7 and D10 (Figure 3B). This pattern of B cell change was found in most individuals (Supplementary Figure 3). Importantly, we identified newly emerged high-ranking IGH lineages at D7 and D10 that were also among the most abundant lineages in samples from other vaccination groups, implying potential functional relevance in the vaccine-induced immune response (Supplementary Figure 1).

These results demonstrated that the TCR repertoire was relatively stable after vaccine boosting, in terms of the frequency dynamic of the most abundant TCR clones, while the IGH repertoire underwent dramatic transformations, explaining the previously illustrated overall differences observed in CDR3 numbers, Shannon index, HCN, and the top 100 cumulative clone frequency.

### Vaccine-Induced IGH Clones Derived From Memory B Cell

Next, we analyzed the IGH isotype compositional changes induced by three different vaccinations. As showed in Supplementary Figure 4A, the percentage of IgM varies among three groups before vaccine boosting at D0 with 5-Helix group showing a significantly higher abundance, indicating that N36 and N51 vaccine possess greater capabilities to induce B cells isotype switch. Correspondingly, a reverse pattern emerged in IgA percentage among the three vaccines (Supplementary Figure 4B). While the IgG percentages were globally low at D0 (~5%), interestingly, the percentages surged across three groups at D7 and tapered to baseline level at D10, with 5-Helix group being the lowest in all three time points (Supplementary Figure 4C). Fold-changes of the three IGH isotypes were further shown, demonstrating the relatively stable IgM and IgA levels, and the significantly gained IgG at D7 in all three vaccination groups (Supplementary Figures 4D–F, note the different y-scales).

**Figure 4:**
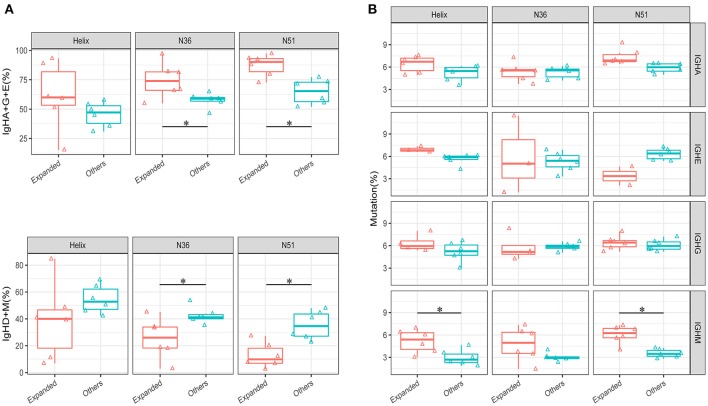
Comparison of the isotype-switch and mutation rates of the lineages at D0. **(A)** The comparisons of percentages (IgH type A, G, and E are shown combined in the upper panel, IgH type M and D shown combined in the lower panel) between vaccination-induced (expanded) lineages and all other lineages in each vaccination group. **(B)** The comparison of average mutation rates for IgHA, IgHE, IgHG, and IgHM between vaccination-induced lineages, and all other lineages in each vaccination group. Comparison were performed using paired Wilcoxon test. *P < 0.05.

An intriguing question that arises from the IGH repertoire dynamics upon vaccination is where do the population of B cells in the expanded IGH lineages come from and what is the nature of these B cells? To answer this question, we first selected the most significantly expanded IGH lineages that were induced by the vaccination boost (see method). We then compared the percentages of IGH isotypes of these lineages with the other unexpanded lineages in the D0 samples. The total percentages of IgA, IgG, and IgE isotypes were much higher in the expanded lineages, especially in group N36 and N51 vaccination groups (Figure 4A), suggesting that these expanded lineages were mostly from the class-switched B cells. We then compared the mutation rates of different isotype B cells. Interestingly, we found a higher mutation rates of IgM B cells in the expanded lineages, indicating the boost also induced affinity maturation of naive cells, apart from the recall of pre-existing memory cells (Figure 4B). Together, these results suggest the selected vaccination-induced IGH clones were mostly from the cell lineages derived from the memory B cell population that pre-dated the vaccine boost.

### Public Expanded TCR Clone Analysis Reveals High Variability Among Individuals After Vaccination

**Figure 5:**
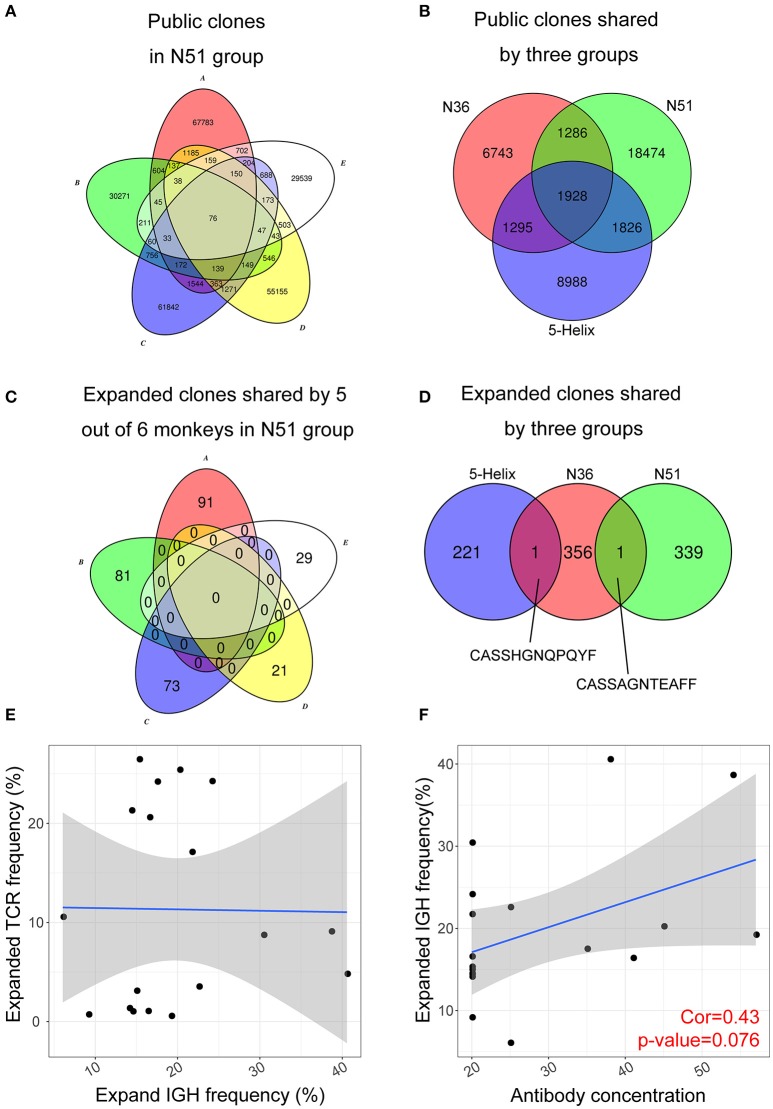
Public expanded TCR clones among vaccination groups. **(A)** Public non-induced TCR clones shared by 5 macaques in the N51 group **(B)**. Public non-induced TCR clones shared by three vaccine groups. **(C)** Public expanded TCR clones shared by 5 maracas in the N51 group **(D)**. Public expanded TCR clones shared by three vaccinated groups. The 6th macaques were not plotted for the simplicity of the graph in A and C **(E)**. The correlation between frequencies of expanded IGH clones and expanded TCR clone **(F)**. The correlation between antibody titer and expanded IGH clone frequency.

A convergent effect might be expected in the TCR clonal evolution upon vaccination of the same or similar antigens, implying that the highly expanded TCRs sharing the same CDR3 sequences emerge in the independently vaccinated animals. It was evident that a large number of public non-vaccine-induced TCR clones were found among individual macaques within the same group (exemplified by N51 in Figure 5A), in addition to their emergence in different groups (Figure 5B). Conversely, we did not find any public expanded clones within each group (exemplified by N51 group in Figure 5C). It was interesting to note that we identified one public clone shared by the 5-Helix and N36 groups, whereas another shared by N36 and N51 groups (Figure 5D). These data imply the possibility that these two TCRs might have originated from the T cell populations that were specifically responsive to the HIV vaccination. The CDR3 sequences (Figure 5D) provide a novel target for therapeutic purposes.

### Antibody Titer Positively Correlates With the Expanded Lineage Frequency

Next, we asked whether the clonal expansion of T cell and B cell were coordinated in individual vaccinated animals as it was possible that expanded B cells were regulated or assisted by the expanded T cell population, or vice versa. Such correlation was not apparent in all three vaccination groups (Figure 5E), suggesting that the expansion of T and B cell lineages developed independently upon vaccination boost. The B cell lineage expansion, however, correlated positively with the previous neutralization assay IC50 results at D7 (Figure 5F), which implies that the induced B cell lineages may develop into antibody-secreting plasma cells during their expansion, producing vast amount of antibodies in response to the vaccination boost.

### Three Vaccines Modulated IGH Repertoires Exhibit Different Features

As B cells that are repeatedly stimulated by antigens in the germinal center (GC) will undergo extensive somatic hypermutation (SHM), each antigen-reactive B cells will ultimately expand and develop into a lineage of plasma cell that generates similar antigens-specific Ig molecules. It has been reported that the vaccine-induced lineages tend to have hypermutated and vastly abundant IGH lineages due to repeated vaccine boosts. Thus, the development of these lineages often reflects the effectiveness of vaccination (36).

**Figure 6:**
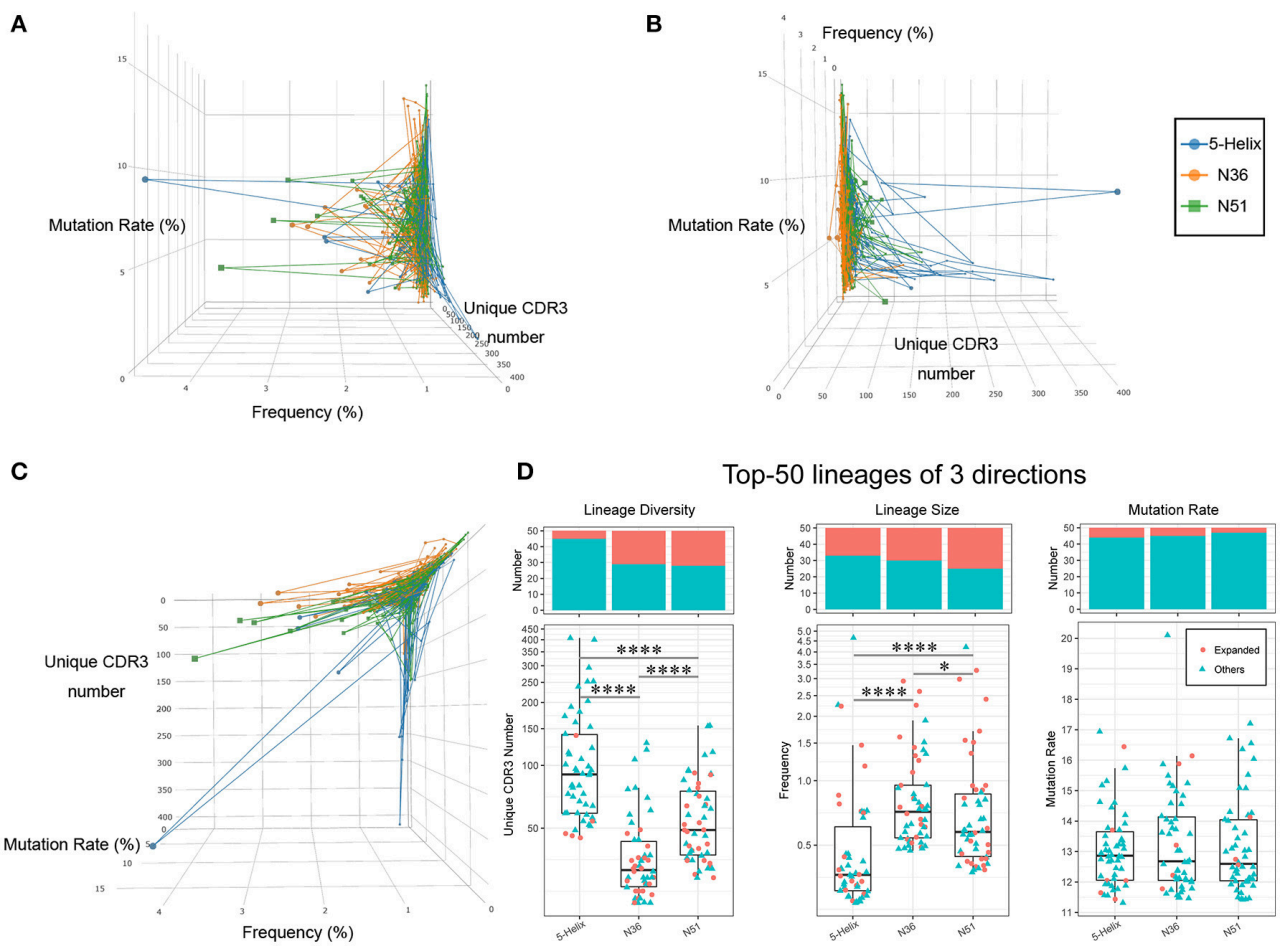
Three vaccines modulate different IGH lineage features. The high abundance lineages (>0.05%) in each sample are plotted in a 3-dimensional space, the 3 axes of which represent the lineage diversity, the lineage size(abundance) and the average mutation rate, respectively. Lineages from different groups are connected and labeled in three different colors (left: blue, orange, green) **(A)**. Projection of lineage diversity axis **(B)**. Projection of lineage size axis **(C)**. Projection of average mutation rate axis **(D)**. Lineage diversity (left panel), lineage size (middle panel), and average mutation rates (right panel) of the top 50 lineages from each vaccination groups are illustrated as boxplots. The histogram plots on the top demonstrates the proportion of vaccination-induced lineages.

We therefore characterized each IGH lineages based on their lineage size (Cumulate frequency of all clones in one IGH lineage), and lineage diversity (the number of CDR3 clone species in one IGH lineage, clones with identical CDR3 amino acids sequence count as one species), and the average mutation rate. The relative levels of these three quantitative features for each IGH lineage (connected dot) are illustrated in a 3-dimensional space (Figures 6A–C). The same projections were further illustrated with two colors representing whether these lineages were vaccination-induced (Supplementary Figures 5A–C). Clearly, the lineages in the three vaccination groups exhibited distinct patterns suggesting specific preferences (Figure 6C). For example, lineages with high lineage diversity but small lineage size were prominent in the 5-Helix group, in comparison to the N36 group. Most of the lineages in the N51 group occupied the space in-between 5-Helix and N36 groups, while some showed big lineage size with slightly more lineage diversity (Figure 6C). Importantly, 5-Helix group includes many lineages with exceptionally high lineage diversity, some of which even surpassed 200. However, these lineages from 5-Helix group showed relatively low mutation rates (<5%) and lineage size (<1%). Intriguingly, these lineages did not belong to the vaccination-induced lineages, suggesting the possible role of indirect immune responses. In contrast, expanded lineages in the N36 and N51 groups exhibited similar pattern with high clonal abundance, moderate mutation rates (5–10%) and medium to high lineage diversity (below 100) (Figures 6A–C, Supplementary Figures 5A–C).

To further illustrate the uniqueness of vaccination-induced lineages, we picked the top 50 lineages of each vaccination group and examined the unique features of distribution in detail. Evidently, lineages in 5-Helix group stood out with significantly higher lineage diversity, while those in the N36 groups displayed the lowest lineage diversity (Figure 6D left panel). Conversely, the lineage size in N36 and N51 group were much greater than those in the 5-Helix group. We also noticed that the vaccination-induced lineages accounted for a significant proportion (25–50%) of the top 50 lineages encompassing all three vaccination groups (Figure 6D middle panel), further affirming the overall effectiveness of vaccination. No meaningful distinctions among the three vaccination groups in terms of average mutation rates were observed (Figure 6D right panel). Taken together, we conclude that the three vaccines could extensively modulate IGH repertoires with different capabilities. Compared with the 5-Helix vaccine, the N51 and N36 vaccines exhibited concordant effects on the induced lineages, with the N51 being superior in inducing IGH repertoire variety. Thus, the sequences found at high levels in response to distinct vaccines present prime targets for the identifications of cross-specific antibodies.

Further, the relevance of these studies can be expanded to human studies for isolating and characterizing BnAbs from HIV-1–infected subjects via the use of computationally derived clonal lineages as templates. This can provide a new path for HIV-1 vaccine immunogen design which can be applicable to many infectious agents, holding a promising future for the construction of vaccines that can drive B cells along unknown but desirable maturation pathways.

## Discussion

Estimating the diversity of a TCR/BCR repertoire using HTS is vital for estimating and evaluating the theoretical size of the repertoire and for tracking changes in clonal populations during the clinical course of infection or vaccination. It may also facilitate quantitative assessment of vaccine-induced B-cell responses and have the potential to provide a predictive indicator of vaccine efficacy. As an example, here we present a detailed antibody analysis of three different vaccines, namely N3, N51, and 5-Helix based on the gp41 pre-hairpin fusion intermediate applied in rhesus macaques. To figure out what changes occurred in the immune repertoire upon vaccine boost, we therefore analyzed the longitudinal vaccine responses using high throughput sequencing (HTS) coupled with *IMonitor* for delineating the evolutionary features of both T cell and B cell repertoires with extreme diversities. These results may help us to understand why three different vaccines performed differently in the neutralization assays.

It is well-known that the repeated stimulation of vaccine antigens can reinforce the memory lymphocytes and trigger these antigen-specific cells to expand. Thus, the proliferation of these cells would promote the expansion of high frequency clones in lymphocyte repertoires. In this study, the TCRB repertoire sequencing results perfectly corroborated such assumption, while the BCR repertoire demonstrated an entirely different pattern of expansion. Seemingly, no unanimous trend was evident based on the basic analysis regarding the diversity and clonality (Figure 2).

Recent studies of neutralizing antibody responses in HIV-infected individuals have used next-generation sequencing (NGS) to study the genetic record of antibody development encoded in peripheral memory B cells (37–39). The germline-encoded antibody segments (V, D, and J) provided critical elements for the interpretation of these data. The currently available heavy chain (HC) gene repertoire for rhesus macaque (30, 40) was obtained by whole-genome sequencing of a single animal with 5 × depth of coverage. A new draft database of VH gene sequences from 10 Indian-origin rhesus macaques was acquired using Illumina deep sequencing to ~50–100 × coverage. Using this new draft database, these studies applied B-cell Ig transcript analysis methods similar to those used previously to interrogate human repertoires (38, 39). Our analysis provides a far broader and a sophisticated longitudinal coverage of vaccine and boost regimens overtime using Hiseq 2000 with paired end reads of 150. Besides, we were aware of the annotation problems caused by the lack of coverage within the rhesus macaque IGH germline genes, thus we brought together the rhesus macaque germline V genes identified in a previous study (30), our novel identified macaque germline V and J genes, together with the IMGT database germline gene into a more accurate gene set to improve our analysis pipeline performance (see Materials and Methods).

Further, previously, immune response to vaccines in humans has been used as a model for studying antibody repertoire in blood samples at well-defined time points (41). Studies with vaccines for influenza and Tetanus Toxoid, have shown dynamic changes in the size and diversity of antibody repertoires before and after antigen stimulation (42, 43). Comparison of post-vaccination responses suggests divergent repertoire properties among individuals, and also within the same individual during vaccine boosting. In order to further understand this phenomenon, we next traced the fate of different clones or lineages of both T and B cells, respectively, during the vaccine boosting process. The original high frequency TRB clones at D7 and D10 were concordantly expanded, however, the IGH repertoire underwent dramatic changes, resulting in infrequent or even undetectable lineages at D0 before the boost becoming the top lineage (Figure 3). It is known that antigen-specific memory B cell or plasma cell formation take place in the secondary lymphoid tissues. Peripheral blood, however, consists of limited proportion of the whole B cell repertoire, thus the peripheral blood B cells are more sensitive to vaccine stimulation.

By analyzing the constant region of IGH, we successfully demonstrated that all three vaccines in this study triggered strong humoral immune response. The IgG subtype B cell proportions in the three groups showed substantial increase despite their original differences, which could be attributed to the differences in the IgG levels among the three vaccines. For instance, the N36 and N51 vaccines are more similar in conformation, thus it is likely that they induced similar responses compared to the 5-Helix vaccine. Together these analyses of the constant region corroborated with our observations on the B cell isotype-switching.

As the antigen-reactive TCR clones and IGH lineages would proliferate during the vaccine stimulation process, we can thus select the vaccine-specific expanded clones or lineages based on the change of frequencies. In order to exclude the false positive clones, we set up stringent cut-off values for this purpose. First, the qualified clones or lineages were of high frequency, avoiding the heterogeneity introduced by the experiment. Second, the D7 and D10 time points after vaccination boost were both taken into consideration to further eliminate random artifacts. Based on these rationales, we obtained the expanded TRB clones and IGH lineages for further analysis. The expanded TRB clones, comparing with the other clones, showed higher similarity to the annotated HIV specific clones recorded in TBAdb—a manually curated database of T- and B-cell receptors with known antigen specificities (Supplementary Figure 6). This result suggests that our selected TRB clones are more likely to be HIV specific. As for the IGH lineages, because the rhesus macaques had been vaccinated several times before the final boost, these expanded lineages were expected to have much more frequent isotype class-switch events. Thus, tracing back the D0 state of the expanded lineages of D7, higher non-IgM and non-IgD percentages were observed in N36 and N51 groups, however, such differences in the 5-Helix group were statistically insignificant (Figure 4A).

We also investigated the mutation rate of expanded lineages at their D0 state. Interestingly, the IgM mutation rate in the expanded lineages was much higher compared to the other lineages, while the IgA and IgG mutation rate showed minimal difference, which was not surprising because any environmental antigens encountered would induce similar spontaneous immune response. Conversely, the higher mutation rate of IgM lineages suggested that most likely these cells were the vaccine-specific memory B cells (Figure 4B).

It is known that the CD4+ T cells are indispensable for the long-lived memory B cell development (44), we therefore also selected the expanded TRB repertoire for analysis. Surprisingly, no correlations were found between the expanded clones and the general public TRB clones, which could be attributed to the heterogeneity of the MHC background. It was also interesting to note that the expanded TRB and IGH lineage frequencies showed no correlation between them (Figure 5E). Considering the statistics of the TCR repertoire, which indicated that T cell also played a pivotal role in the vaccine boost responses, we hypothesized that the T and B cell responses to these vaccines were relatively independent, requiring more detailed studies.

Because of the repeated modulation of HIV vaccine to the B cell repertoire, vaccine reactive B cells evolved by somatic hypermutation, and gradually developed into larger lineages with many highly abundant BCR clones sharing similar sequences with relatively high mutation rate. Thus, by analysis of lineage abundance, unique clone numbers and mutation rates, we found that the lineages of three vaccination groups showed distinct patterns, which echoes the future expanded lineages. The N51 and N36 vaccine performed superior in inducing the lineage size expansion, while 5-Helix vaccine induced more low-abundance clones in the expanded lineages (Figure 6 and Supplementary Figure 5). together with the lineage isotype switch and mutation analyses showed in Figure 4, our results somehow corroborated with the neutralizing assays where N51 and N36 showed higher HIV-1 neutralizing ability than 5-Helix.

In some cases, the initial antibodies binding to HIV-1 gp41 in acutely infected subjects are polyreactive and highly mutated (45). It was therefore speculated that these antibodies might be generated by the memory B cells that interacted with non-HIV-1 antigens (45, 46), Nevertheless, the production of considerable amount of high affinity broadly neutralizing antibodies (bnAbs) still requires repeated reinforcements to the immune memory against the pathogen. Also, the development of these bnAbs and immune status can only be monitored and traced through the high-throughput sequencing methodology. The features of the repertoire modulated by different vaccines were extensively compared in this study, however, more layers of information could be added in the future to enable a more specific and functionally relevant selection of HIV-1-specific B cell lineages. For instance, longitudinal studies (47) based on the reliable vaccine-specific B cell lineages combined with the use of methodology described in the current study would be excellent in delineating T and B cell repertoires and monitoring responses to anti-HIV vaccines.

Through the analysis of T and B cell repertoires in response to 3 different envelope-based anti-HIV vaccines in Rhesus macaques, this study demonstrates the utility of computationally derived clonal lineages as templates may provide a new paradigm that may assist the designing of HIV vaccine immunogen, which is sorely needed for creating an effective vaccine. This computational approach may have broader implications for many infectious pathogens other than HIV, and may hold considerable promise for the construction of new generation of vaccines, which could drive and guide B cells to more desirable maturation pathways for the creation of effective HIV vaccines.

Overall, our results demonstrated that the comprehensive analysis method for T and B lymphocytes repertoires greatly facilitated monitoring of the immune status dynamics during vaccine boost. Undoubtedly, the combination of the experimental methods including fluorescence activated cell sorting, single-cell sequencing and new bioinformatics tools will additionally provide more detailed information with higher resolution in cell-to-cell networking, thereby optimizing and expediting the HIV vaccine development. Our study represents the first attempt in the characterization of antibody repertoire sequence diversity in vaccinated macaques with anti-HIV vaccines, which demonstrates the utility of the HTS in elucidating entire antibody repertoires at the sequence level from individual animals. Since macaques are being used as models for testing human anti-HIV vaccines, these data provide significant insights into understanding the primate immune system function in response to these vaccines, and will assist in future engineering and designing of these vaccines for human use.

There is enormous potential of immune repertoire studies in clinical applications (18, 20, 22, 48), more work is needed in identifying and incorporating the well-defined dynamic sequence changes and molecular signatures that may provide reliable and durable clinical outcomes. However, questions remain on how the alterations in the immune repertoire are intrinsically linked to the severity of infections and its relationship with the CDR3 sequence-based manifestations, and how the abundance of protective immunoglobulins or T cell from a given sequence library can be predicted. Further advances in High throughput technologies will ultimately provide a clear blueprint of the adaptive immune response, which will facilitate a rational design of various immunotherapies for infection, including HIV.

## Ethics Statement

The research was prospectively reviewed and approved by a duly constituted ethics committee (the institutional review board on bioethics and biosafety of BGI ethical approval).

## Author Contributions

I-MW and XLiu designed the study and supervised research. LW performed research, analyzed data, and wrote the paper. WZ analyzed the data. LL collected the specimens and clinical information, and performed the experiments. I-MW and JJ contributed samples and performed the experiments. All authors contributed to the preparation of the manuscript and approved the submission in its current form.

### Conflict of Interest Statement

The authors declare that the research was conducted in the absence of any commercial or financial relationships that could be construed as a potential conflict of interest.
