# Corrigendum: A Comprehensive Analysis of the T and B Lymphocytes Repertoire Shaped by HIV Vaccines

**DOI:** 10.3389/fimmu.2018.02931

**Published:** 2018-12-14

**Authors:** Longlong Wang, Wei Zhang, Liya Lin, Xiao Li, Nitin K. Saksena, Jinghua Wu, Shiyu Wang, Joseph G. Joyce, Xiuqing Zhang, Huanming Yang, Jian Wang, I-Ming Wang, Xiao Liu

**Affiliations:** ^1^BGI-Education Center, University of Chinese Academy of Sciences, Shenzhen, China; ^2^BGI-Shenzhen, Shenzhen, China; ^3^China National GeneBank, BGI-Shenzhen, Shenzhen, China; ^4^Merck & Co., Inc., Kenilworth, NJ, United States; ^5^James D. Watson Institute of Genome Sciences, Hangzhou, China

**Keywords:** HIV, vaccine, T cell receptors repertoire, B cell receptors repertoire, gp41

In the original article, there was a mistake in Figure 3. The two Circos diagrams in Figure 3 were imbedded upside down, which means that Figure 3A was supposed to be Figure 3B and vice versa. The corrected Figure 3 appears below.

**Figure 3:**
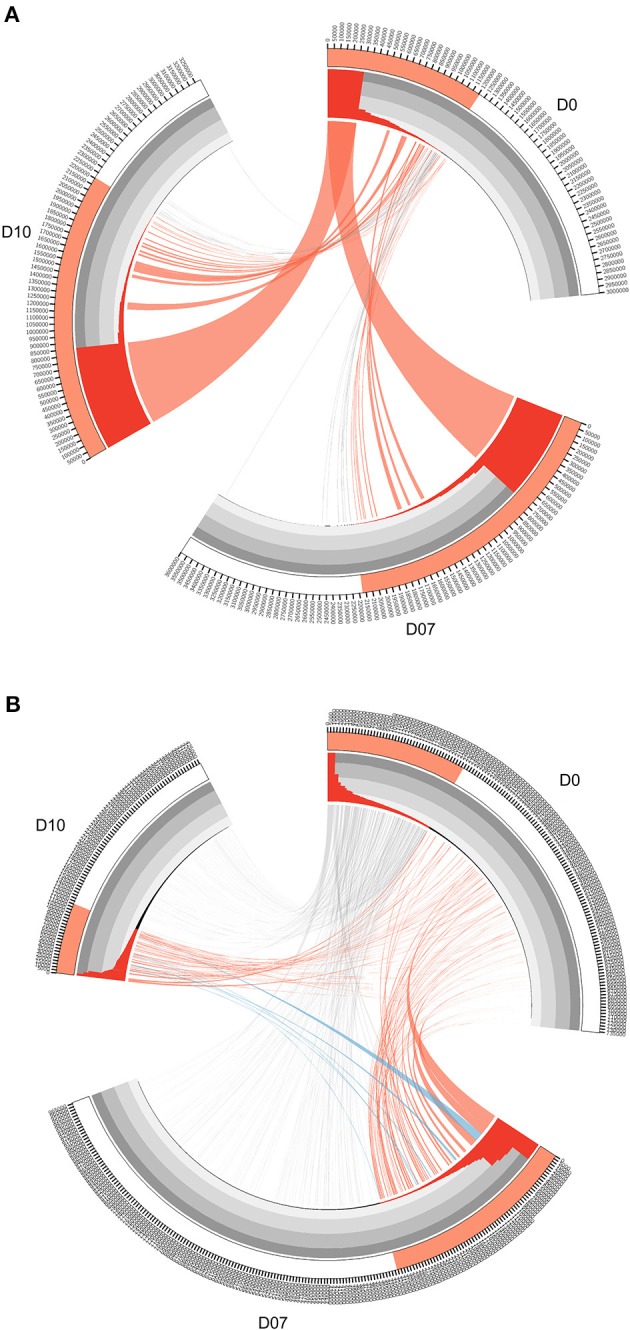
Circos diagrams of pattern change of TRB clone frequency and IGH lineage frequency during the time course of final boost of rhesus macaques A5R069 from the N51 group. **(A)** Clones were sorted by their frequency at three different time points, the clone number of each time point is ticked on the outer lane. High abundance clones (>0.01%) are marked in red. The inner histogram lane shows the frequency of each clone with the height of each bar representing the relative abundance. The width of both the histogram and ribbon represents the frequency of each clone. Ribbons connect the same TCR clones at different time points. The ribbons connecting the high abundance clones at D7 and D10 that showed increase in frequency of more than 1-fold compared to D0 were labeled in red, those connecting the clones showing 50% decrease in frequency were labeled in gray, and those connecting the newly emerged clones that have high abundance (>0.01%) were labeled in blue **(B)**. IGH lineages were plotted similarly as in **(A)**, with the exception that red ribbons connecting high abundance lineages (>0.05%) at D7 and D10 that showed increase in frequency by more than 3-fold compared to D0.

The authors apologize for this error and state that this does not change the scientific conclusions of the article in any way. The original article has been updated.

## Conflict of Interest Statement

The authors declare that the research was conducted in the absence of any commercial or financial relationships that could be construed as a potential conflict of interest.

