# Supplementary material for: A Comprehensive Analysis of the T and B Lymphocytes Repertoire Shaped by HIV Vaccines
Source: Front Immunol. 2018 Sep 26;9:2194. doi: 10.3389/fimmu.2018.02194 (PMC6168627; doi:10.3389/fimmu.2018.02194)
Supplement: Supplementary file 7 [file Data_Sheet_1.pdf]

Supplementary Materials

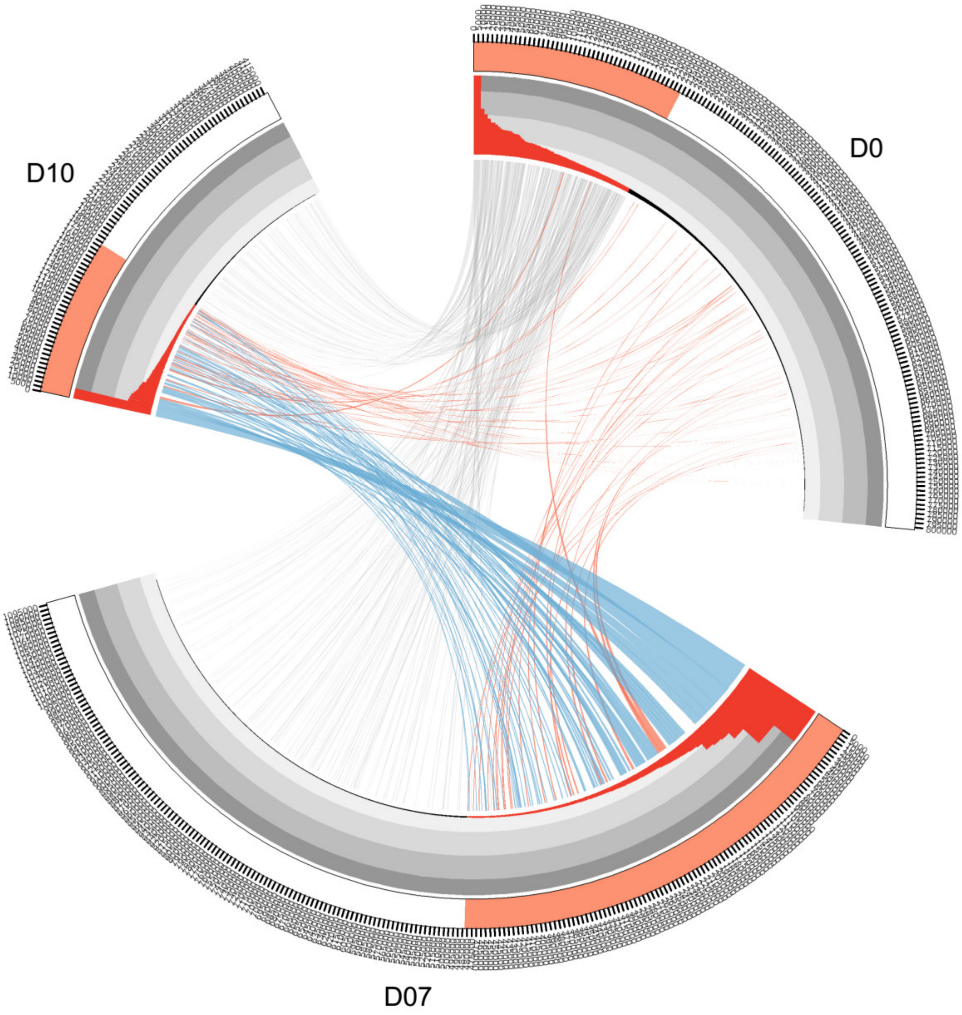

Supplementary Figure 1: Circos diagrams of pattern change of IGH lineage frequency during the time course of final boost of rhesus macaques 05D131 from N51 group.

(A) IGH lineages were sorted by their frequency at the three time points, the clone number of each time point is ticked on the outer lane. High abundance lineages(>0.05%) are marked in red. The inner histogram lane shows the frequency of each clone with the height of each bar representing the relative abundance. The width of both the histogram and ribbon represents the frequency of each clone. Ribbons connect the same IGH

lineages at different time points. The ribbons connecting the high abundance lineages at D7 and D10 that had increase in frequency for more than 3-folds compared to D0 were labeled red, while those connecting the lineages decreased more than 50% in frequency were labeled grey, and those connecting the newly emerged clones that have high abundance(>0.05%) were labeled blue.

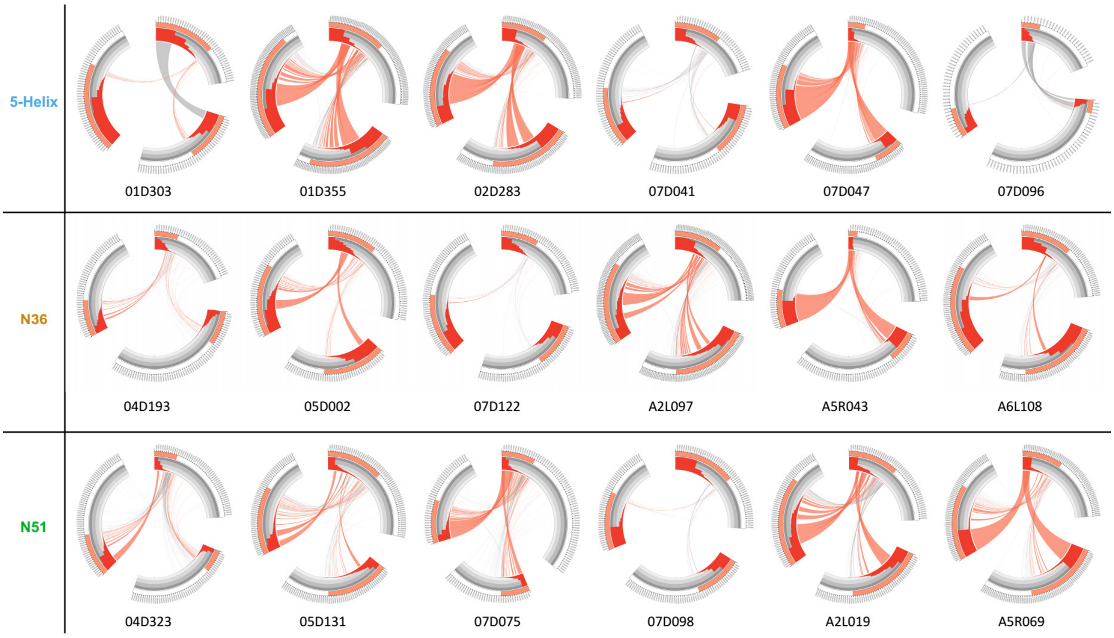

Supplementary Figure 2: Circos diagrams for of pattern change of TRB lineage frequency during the time course of final boost for all of the eighteen rhesus macaques.

Circos diagrams of pattern change of TRB lineage frequency during the time course of final boost for all of the eighteen rhesus macaques from the three different vaccination groups (5-Helix, N36, N51), each one of the circos diagrams was plotted consistent with Figure 3A.

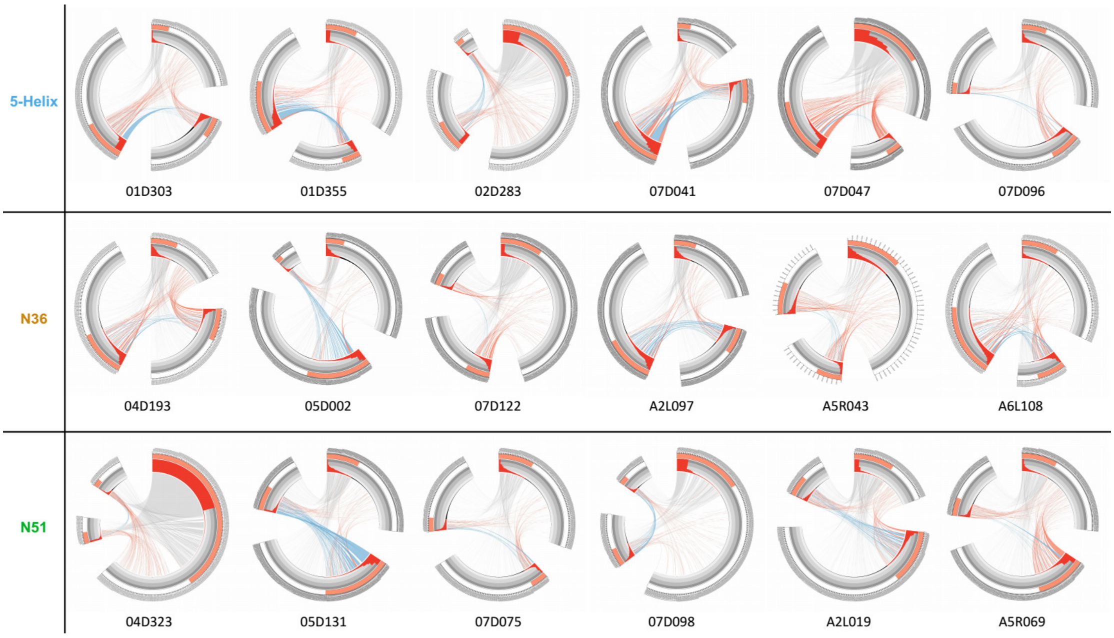

Supplementary Figure 3: Circos diagrams for of pattern change of IGH lineage frequency during the time course of final boost for all of the eighteen rhesus macaques.

Circos diagrams of pattern change of IGH lineage frequency during the time course of final boost for all of the eighteen rhesus macaques from the three different vaccination groups (5-Helix, N36, N51), each one of the circos diagrams was plotted consistent with Figure 3B.

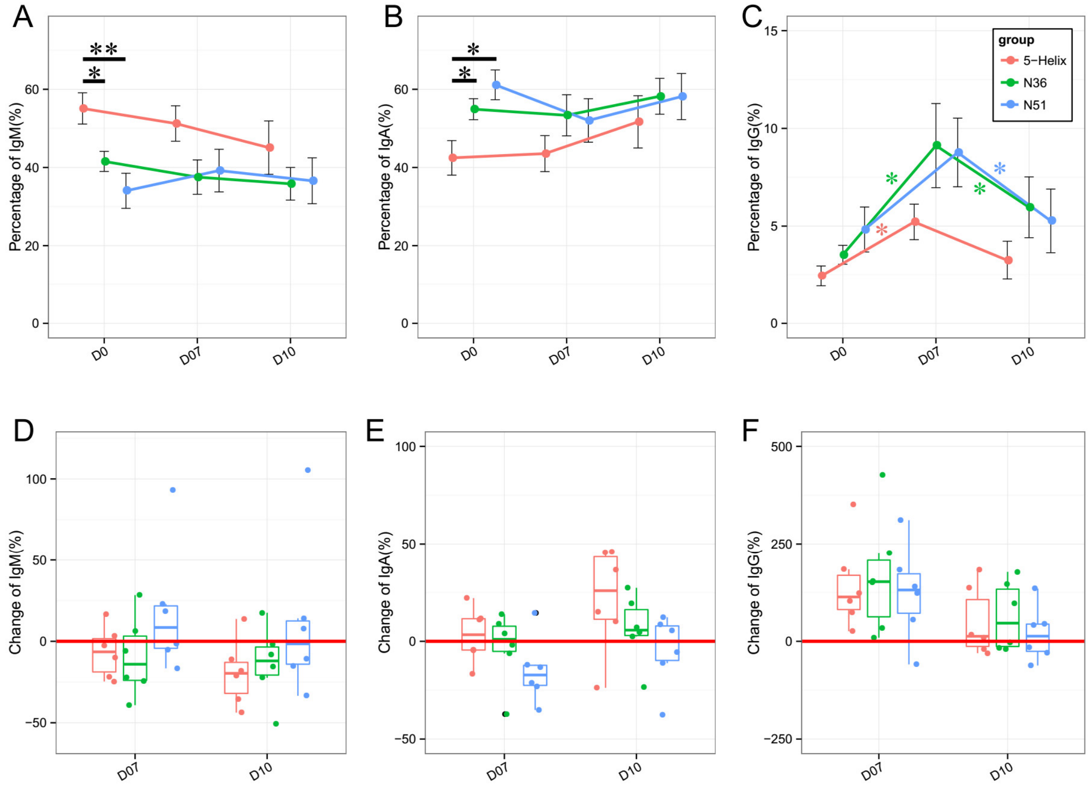

Supplementary Figure 4: Representation of isotype component feature of IGH repertoire in three vaccination groups.

(A-C) Isotype percentages of IgM, IgA and IgG, respectively, at the three different time points. Each point represents the mean value of a 6-macaque group at one time point with a bar of ±SEM. Red, green and blue color represent 5-Helix, N36 and N51, respectively(Black * P<0.05, **P<0.01, Wilcoxon test, unpaired (n=6))(Colored * P<0.05, **P<0.01, Wilcoxon test, paired (n=6))(D-F). Box plots depicting fold changes of isotype percentages at D7 and D10 in comparison to D0, with red, green and blue color representing 5-Helix, N36 and N51, respectively.

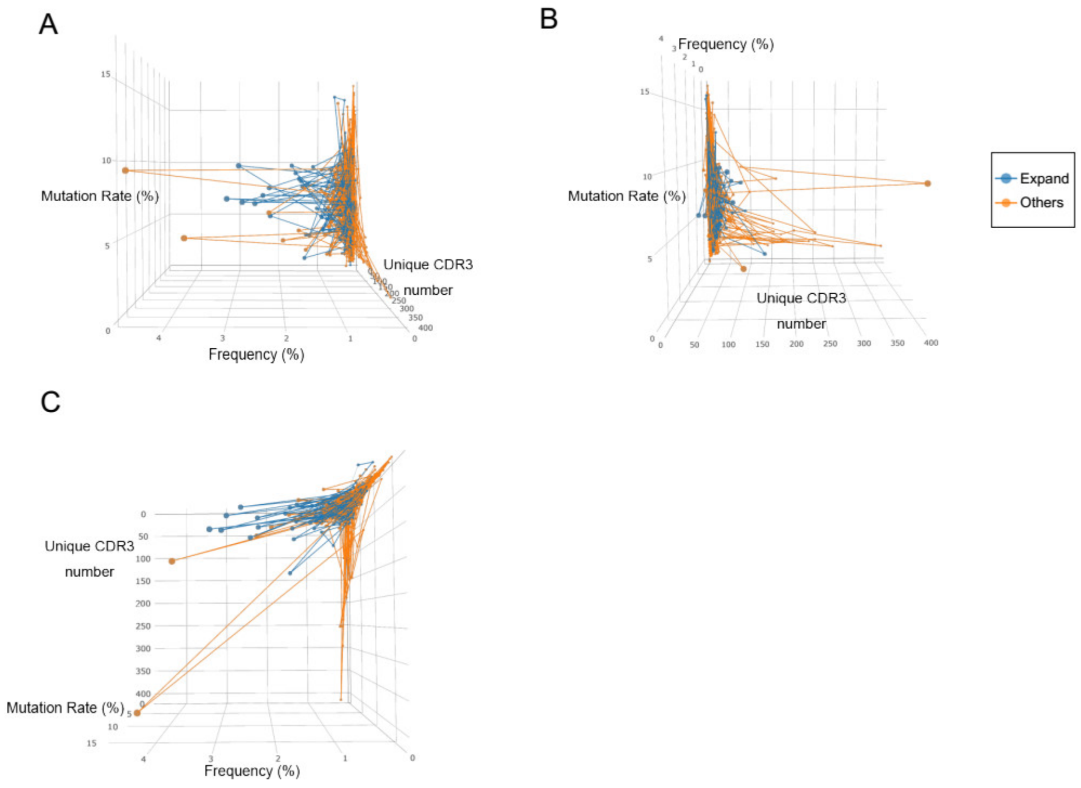

Supplementary Figure 5: Expanded lineages exhibit characteristic features and patterns.

The high abundance lineages(>0.05%) in every sample are plotted in a 3-dimension space whose 3 axes represents the lineage-specific cdr3 numbers, the lineage frequency and the lineage mutation rate respectively. Expanded lineages are marked and lined up in blue color, other lineages are marked in orange.**(A)** View of lineage unique CDR3 number axis direction.(B) View of lineage frequency axis direction.(C) View of lineage mutation rate axis direction.

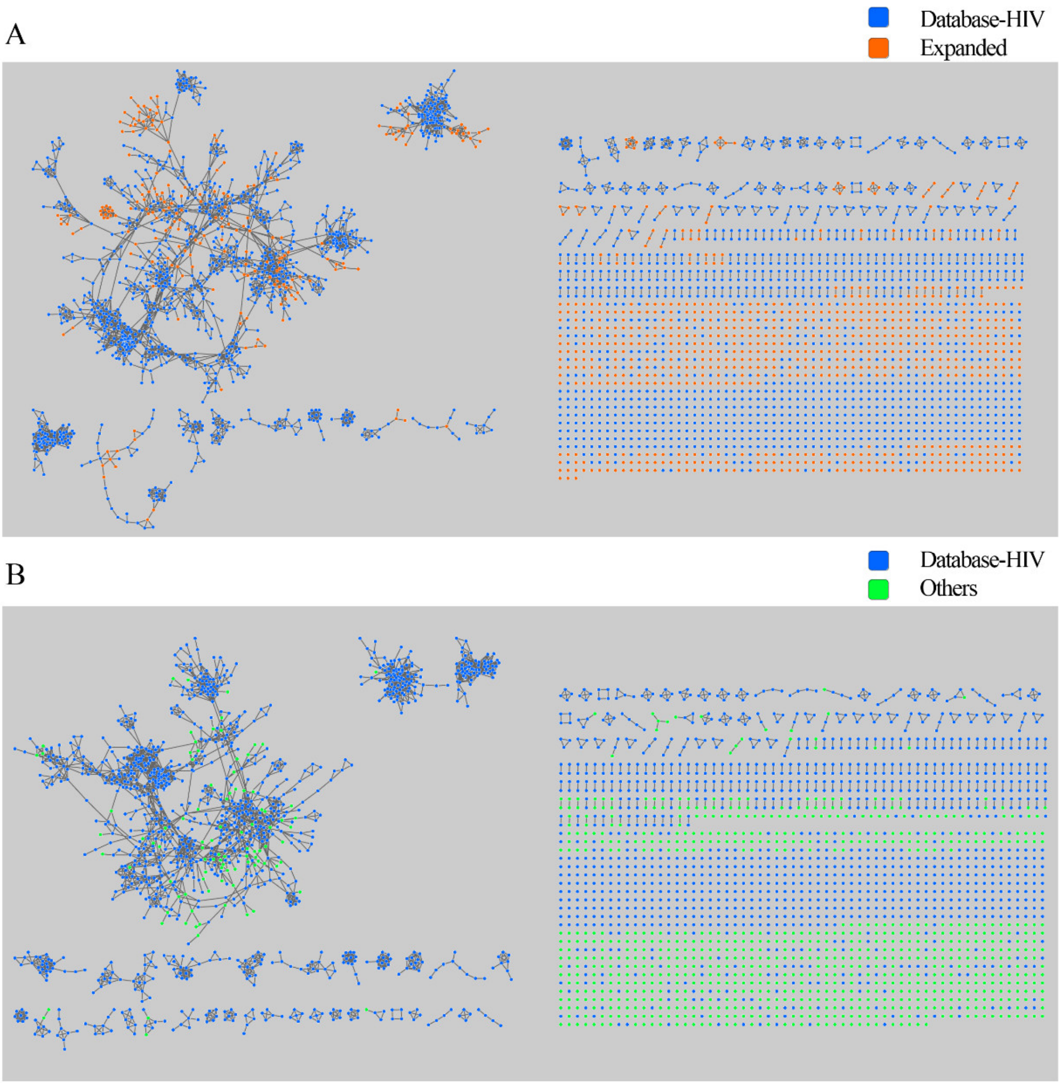

Supplementary Figure 6: Expanded TRB clones clusters better with Database HIV specific clones.

(A) A total of 918 expanded TRB clones are cluster with database HIV specific clones, each node represents one TRB clone, similar clones (Levenshtein distance less than 2) are lined up with grey edges. **(B)** A total of 918 randomly selected other TRB clones are cluster with database HIV specific clones, each node represents one TRB clone, similar clones (Levenshtein distance less than 2) are lined up with grey edges.
